# Low Serum 25-Hydroxy Vitamin D (25-OHD) and Hepatic Encephalopathy in HCV-Related Liver Cirrhosis

**DOI:** 10.1155/2021/6669527

**Published:** 2021-02-12

**Authors:** Mohamed Abd Ellatif Afifi, Ahmed Mohamed Hussein, Mahmoud Rizk

**Affiliations:** Internal Medicine Department, Hepatology and Gastroenterology Unit, Benha University, Egypt

## Abstract

**Background:**

Patients with liver cirrhosis experience a large variety of metabolic disorders associated with more hepatic decompensation. Hepatic encephalopathy (HE) is a significant complication in liver cirrhosis patients, presenting a wide spectrum of neuropsychological symptoms. A deficiency of 25-hydroxy vitamin D (25-OHD) in the general population is associated with a loss of cognitive function, dementia, and Alzheimer's disease. *Aim of the Study*. Our study aims to check the relationship between low serum 25-OHD and HE in patients with HCV-related liver cirrhosis and assess its link with patient mortality. *Patients and Methods*. This study was observationally carried out on 100 patients with HCV-related liver cirrhosis. The patients were divided into 2 groups: Group A—included 50 HCV-related cirrhotic patients with HE, and Group B—included 50 HCV-related cirrhotic patients without HE. Assessment of disease severity using the end-stage liver disease (MELD) model and Child Turcotte Pugh (CTP) scores were done, and 25-OHD levels were measured. Comparison of vitamin D levels in different etiologies and different CTP categories was made using one-way ANOVA. Pearson's correlation between the level of vitamin D and other biomarkers was applied.

**Results:**

There was a statistically significant Vitamin D level difference between the two groups. A lower level of vitamin D was observed in the HE group where the severe deficiency was 16%, while it was 6% in the other group and the moderate deficiency was 24% in HE group as compared to 10% in the other group. The insufficient vitamin D level represented 46% of the non-HE group while none of the HE group falls in this category. Vitamin D level was statistically higher in Grade 1 HE than in Grade 2 which is higher than in Grades 3 to 4. Vitamin D level was also significantly higher in those who improved from HE as compared to those who died.

**Conclusion:**

The lower levels of 25-OHD were associated with the higher incidence of HE in cirrhotic HCV patients. The worsening vitamin D deficiency was associated with increased severity of the liver disease, so vitamin D may be considered a prognostic factor for the severity of liver cirrhosis and high mortality rate in HE patients.

## 1. Introduction

Hepatitis C virus (HCV) infection is a global public health problem. More than 185 million human beings (3% of the world's population) are infected. Nearly seventy-five percent of HCV patients are living in middle to low-income countries [1, 2]. Unfortunately, Egypt is one of the countries having the highest global burden of HCV infection with a prevalence of around 4.6% to 6.8% [3].

HCV infection is considered the leading cause of chronic hepatitis, cirrhosis, and hepatocellular carcinoma (HCC). About fifty-five to fifty-eight percent of HCV patients develop fibrosis and cirrhosis and may deteriorate to decompensated liver cirrhosis, HE, and hepatocellular carcinoma [4].

In patients with HCV cirrhosis, ascitic fluid infection as in spontaneous bacterial peritonitis (SBP) is common and is considered a significant risk factor for circulatory dysfunction and the development of hepatorenal syndrome (HRS) [5–7]. Moreover, disturbance in blood flow to the brain is integrated into the pathogenesis of HE and more likely linked to alterations in the ammonia level and inflammatory status [8].

HE describes a complex variety of neuropsychiatric symptoms ranging from subclinical changes to coma. It is classified according to West Haven criteria into minimal (covert) or overt encephalopathy [9]. Up to fifty-five percent of patients with the chronic hepatic disease have been reported with HE [10]. Symptoms include impaired memory and motor control and decreased levels of energy [11]. HE features can be similar to signs seen in dementia patients [12]. The etiology and pathogenesis of HE are complex and multifactorial, including impaired metabolism of ammonia, dysbiosis that causes inflammation in the intestine and liver [13], decreased levels of circulating branched amino acids [14], abnormalities in electrolyte levels [15], and changes in zinc and manganese levels [16].

Importantly, after treatment, the symptoms and signs of HE can often be significantly reversed, confirming that it is a functional and not a structural cause of cognitive impairment [17]. HE development presents major challenges for patients and their carers [18]. Until recent times, lactulose has been the major cornerstone of HE management and is still been used as first-line management to control symptoms of chronic HE and to reverse the symptoms of episodes of acute HE [19]. The introduction of rifaximin decreased HE rates and duration of hospital admission and frequency due to OHE and also improved the quality of life for the patients and their carers [20]. Patients with recurrent episodes of OHE may encounter persistent and cumulative deficits in memory and the ability to learn [21].

Vitamin D is considered a multifunctional steroid hormone with many actions. Little about Vitamin D's function is actually understood. It is becoming more clear that vitamin D is not only engaged in calcium homeostasis and bone metabolism but it also has multiple biological actions mediated by vitamin D receptors (VDR) [22] present in even more than thirty tissues [23] including the kidneys, brain, intestine, pituitary, parathyroid gland, prostate, breast, heart muscle, skeletal muscles, hepatic cells, immune system, and endothelial cells [24–28].

Vitamin D is obtained from certain food stuffs and exposure to ultraviolet light. Activation of vitamin D is done through two steps; the first is through hydroxylation of cholecalciferol into the 25-OHD metabolite that occurs in the liver, which is the major circulating metabolic form of vitamin D. It circulates bound to the vitamin D-binding protein (DBP) with a 15-21 day half-life. The second is through an activation process for 1,25 dihydroxy vitamin D which occurs mainly in the kidney and to a lesser extent in a range of all other tissues such as the bone, mammary gland, brain tissue, monocytes, parathyroid gland, and placenta. This active metabolite has a shorter 10–20-hour half-life. Accordingly, the level of vitamin D is commonly assessed by measuring circulating 25-OHD [29, 30].

Vitamin D is an immune modulator and is considered as an anti-infective agent [31]. Clinical associations among vitamin D status and global and specific areas of cognitive function are increasing, and vitamin D deficiency may also be associated with both depression and schizophrenia [32, 33]. Furthermore, in some areas of cognitive function, vitamin D deficiency is associated with low mood and impairment of physical performance [34] as well as with a rapid decrease in cognitive function [35].

Most neurons have VDR proteins, including brain glia [36]. Key areas of cognition are the hypothalamus and the brain cortex [37]. The presence of both the VDR protein and vitamin D metabolites in these brain areas is an indication that the vitamin D system is involved in the brain's normal function [38].

As the liver is responsible for the first step of hydroxylation of vitamin D into 25-OHD, the levels decrease with more advanced liver disease. Decreased vitamin D level has been reported in about 92 percent of patients with chronic liver disease (CLD), with a severe 25-OHD deficiency in at least one-third of them [39, 40]. Also, it is noticed that increased overall mortality in cirrhotic patients is related to a more deficient 25-OHD levels [41].

Our research is aimed at studying the relationship between low serum 25-OHD and HE in HCV-related liver cirrhosis patients and assessing its link to their mortality.

## 2. Patients and Methods

### 2.1. Study Design

This is a hospital-based noninterventional, prospective, observational study carried out in the internal medicine department (ward–ICU unit) of Benha University Hospitals, Egypt.

### 2.2. Study Population and Participant Recruitment

**Figure 1:**
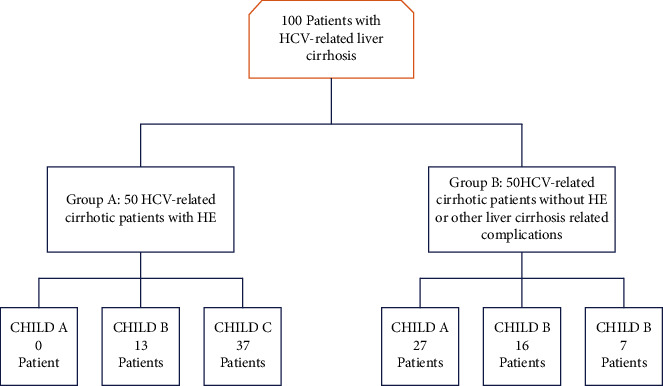
Patient selection flowchart (Afifi, Hussein, and Rizk, 2020).

The study was conducted on 100 patients with HCV-related liver cirrhosis who were divided into 2 groups: Group A—including 50 HCV-related cirrhotic patients with HE, and Group B—including 50 HCV-related cirrhotic patients without HE or other liver cirrhosis-related complications (Figure 1).

#### 2.2.1. Inclusion Criteria

The following are the inclusion criteria:

- Patients of both sexes aged 18 years or more
- Eligible patients who had a diagnosis of liver cirrhosis secondary to HCV infection
- Diagnosis of liver cirrhosis based upon clinical, laboratory, and U/S features

#### 2.2.2. Exclusion Criteria

The following are the inclusion criteria:

- Age < 18 years
- Patients with liver cirrhosis secondary to causes other than HCV infection
- Patients with a history of vitamin D supplementation in the previous 2 months
- Patients with a history of a wide variety of medications including that lead to low vitamin D levels (e.g., antifungal, anticonvulsant, or glucocorticoid medications).
- Patients with vitamin D deficiency due to other causes like (limited sunlight exposure, CKD, IBD, malabsorption syndrome, obstructive jaundice, lymphomas, or hyperparathyroidism)
- Patients with HCC

### 2.3. Consent

Written informed consent was obtained from all individual participants included in the study. For patients with altered consciousness, informed consent was obtained from their first-degree relatives.

### 2.4. Patients Were Subjected to

The patients were subjected to the following:

- A detailed history taking and a clinical examination for stigmata of CLD including ascites and presence of HE; assessment of the severity of patients presenting with HE done according to West Haven criteria [42]
- An assessment of disease severity using a model for end-stage liver disease (MELD) [43] and CTP scores [44]
- Biochemical parameters (CBC, albumin, bilirubin, INR, serum creatinine, serum sodium, serum potassium, and alpha fetoprotein L3)
- Pelvi-abdominal U/S to detect radiological features of cirrhosis (irregular surface, coarse texture, and attenuated hepatic veins) and ascites and to exclude hepatic focal lesions
- A diagnostic abdominal paracentesis of patients with ascites to obtain an ascetic fluid sample for polymorphonuclear neutrophil (PMN) count and ascitic fluid culture for diagnosis of SBP

A combination of the BacT/Alert Microbial Detection System with the VITEK 2 system (bioMérieux, France) was used to achieve rapid bacterial identification and susceptibility testing in the diagnosis of SBP [45, 46].

### 2.5. Measuring 25-OH Levels

25-OH levels were measured using the Diasorin RIA® assay technique. 25-OH vitamin D status was defined as sufficient (>75 nmol/L), insufficient (50-75 nmol/L), mild deficiency (25-50 nmol/L), moderate deficiency (12.5-25 nmol/L), and severe deficiency (<12.5 nmol/L) [47, 48].

### 2.6. Statistical Methods and Data Analysis

Descriptive statistics for the two groups were presented as the mean and standard deviation for numerical variables and as frequencies and cumulative frequencies for categorical variables. A comparison of differences between the two groups was done using the independent *t*-test for numerical variables, and using the chi-square test as well as Fisher's exact test for categorical variables. The comparison of vitamin D levels in different HE etiologies and different CTP categories was made using one-way ANOVA. The comparison of 25-OHD levels in different SBP categories and different HE grades was made using the Kruskal-Wallis Test. The correlation between the level of vitamin D and other biomarkers was made using Pearson's correlation.

## 3. Results

### 3.1. Patient Characteristics and Biochemical Analysis

Among the enrolled 100 patients, Group A included 50 patients having HCV-related cirrhosis complicated with HE (13 patients Child B and 37 patients Child C) and Group B included 50 patients having HCV-related cirrhosis without HE (27 patients Child A, 16 patients Child B, and 7 patients Child C) (Figure 1). Baseline characteristics were compared in both groups showing nonsignificant difference regarding age. BMI was higher for the HE group (mean = 28.7, SD = 3.5) than the non-HE group (mean = 25.7, SD = 3.1). The difference of potassium level and WBCs count in both groups was not significant. There was a significant difference between both groups in other variables. MELD score and CTP score were higher in the HE group (21.8 ± 5.6) and (11.8 ± 2.4), respectively, versus (12.6 ± 5.9) for MELD and (7.0 ± 2.1) for CTP in the non-HE group. Moreover, bilirubin, creatinine, and INR were higher in the HE group, while albumin, sodium, hemoglobin, and platelets were lower in the HE group (Table 1).

### 3.2. Comparing Both Groups as regards Other Categorical Variables (Table 2)

#### 3.2.1. Sex

There was no significant difference between both groups regarding sex (group 1 included 38 males and 12 females, while group 2 included 33 males and 17 females with *P* value = 0.271).

#### 3.2.2. CTP Classification

None of the HE group was classified as A, while 54% of the non-HE group were classified as A, and 17% of this group were classified as C while 74% of the HE group were classified as C, and this difference was statistically significant.

#### 3.2.3. Ascites

6% of the HE group had no ascites as compared to 54% of the non-HE group while 54% of the HE group had poorly controlled ascites as compared to 20% of the non-HE group and this difference was statistically significant.

#### 3.2.4. Other Variables

Comparing both groups regarding SBP, HRS, and hematemesis and/or melena (H/M) was statistically significant. The entire non-HE group did not have SBP, HRS, or H/M. Thirty percent of the HE group prognosis was death as compared to 0% in the non-HE group.

### 3.3. Vitamin 25-OHD Levels in Both Groups

A higher degree of 25-OHD deficiency was observed in the HE group where the severe deficiency was 16%, as compared to 6% in the other group, and the moderate deficiency was 24% as compared to 10% in the other group. The insufficient 25-OHD level represented 46% of the non-HE group while none of the HE group falls in this category. Differences in 25-OHD levels between both groups were highly significant (Table 3).

### 3.4. Etiology of HE

The most common etiology of HE was the SBP (32%), followed by H/M (20%), HRS (18%), and hypokalemia (14%), while others' etiologies represented 16% (Table 4).

### 3.5. Comparing 25-OHD Levels in Different HE Etiologies

**Figure 2:**
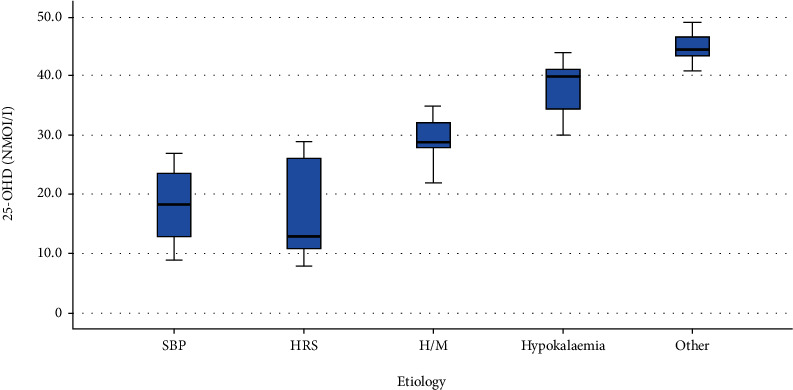
Comparison of 25-OHD levels in different HE etiologies (Afifi, Hussein, and Rizk, 2020).

The levels of 25-OHD in the SBP and HRS groups were less than those in the H/M group which were less than those in the hypokalemia as shown in (Table 5, Figure 2).

### 3.6. Comparing 25-OHD Levels in Different SBP Categories, Child Classes, and the Correlation with MELD Score

**Figure 3:**
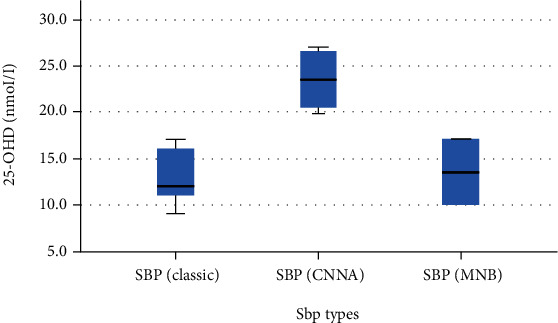
Comparison of 25-OHD levels in different SBP categories (Afifi, Hussein, and Rizk, 2020).

**Figure 4:**
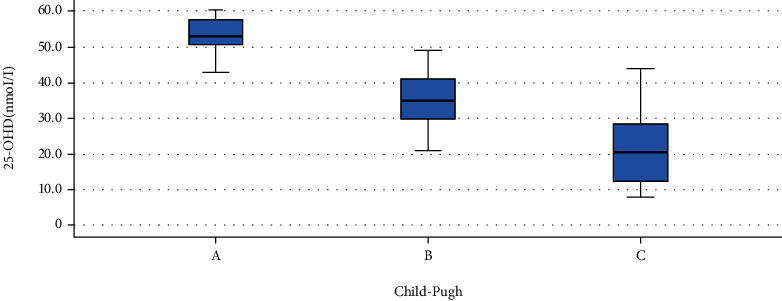
Comparison of 25-OHD in different CTP categories (Afifi, Hussein, and Rizk, 2020).

**Figure 5:**
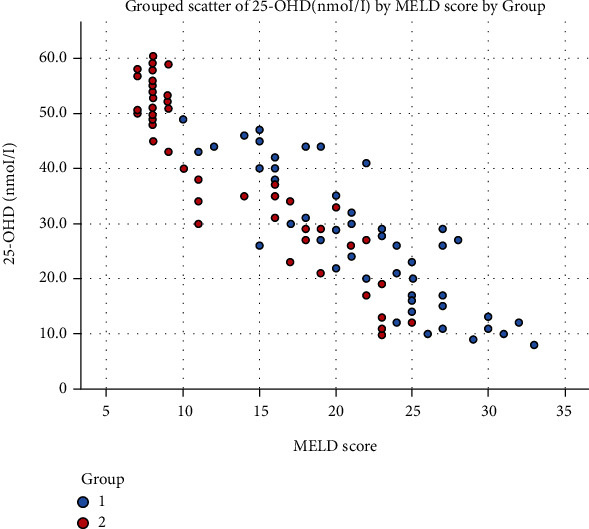
Grouped scatter plot showing the correlation between the 25-OHD level and MELD score (Afifi, Hussein, and Rizk, 2020).

The levels of 25-OHD in the SBP (classic and monobacterial (MNB) groups were less than the culture-negative neutrocytic ascites (CNNA)) group, and this difference was statistically significant (Table 6 and Figure 3). The level of 25-OHD in Child A was higher than Child B and Child B was higher than Child C (Table 7, Figure 4). There was a strong negative correlation (r = -0.91) between the 25-OHD level and the MELD score (Table 8, Figure 5).

### 3.7. Comparing 25-OHD Levels in Different HE Grades

**Figure 6:**
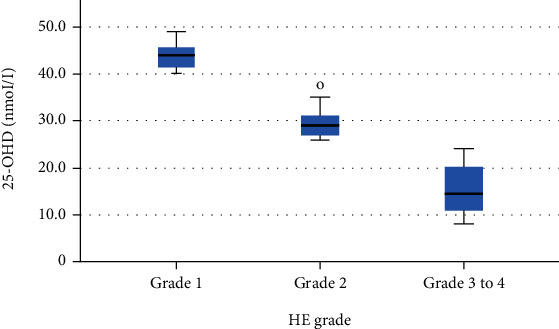
Comparison of 25-OHD level in different HE grades (Afifi, Hussein, and Rizk, 2020).

The level of 25-OHD in Grade 1 was higher than that in Grade 2 which was higher than that in Grades 3 to 4, and this difference was statistically significant (Table 9, Figure 6).

### 3.8. Comparing 25-OHD Levels in relation to Prognosis

**Figure 7:**
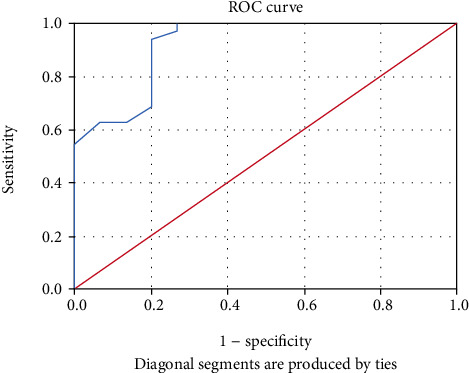
ROC curve for the 25-OHD level to predict improvement in HE group (patients who have recovered and did not die) (Afifi, Hussein, and Rizk, 2020).

Comparing 25-OHD levels between those who died (mean 15.1 ± 7.5) and those improved in the HE group (mean 32.5 ± 9.5) revealed that 25-OHD levels were significantly higher for those who improved as compared to those who died (Table 10). ROC curve for 25-OHD levels showed the cut-off value 18.5 (nmol/L) to predict improvement in the HE group (patients who recovered and did not die) with a sensitivity of 94% and a specificity of 80% (Figure 7). By using 18.5 (nmol/L) as a cut-off level for 25-OHD for survival in the HE group, it showed a sensitivity of 94% and a specificity of 80% (Table 11).

### 3.9. Correlation between 25-OHD Level in the HE Group and Other Variables

There was a strong negative correlation between bilirubin (*r* = −0.85, *P* = <0.001), creatinine (*r* = −0.55, *P* = <0.001), INR (*R* = −0.65, *P* = <0.001), and potassium (*r* = −0.39, *P* = <0.005) levels and serum 25-OHD level. Strong positive correlation was found between albumin (*r* = 0.81, *P* = <0.001) and sodium (*r* = 0.71, *P* = <0.001) levels and serum 25-OHD level. No correlation was found between 25-OHD level and hemoglobin level, WBCs, or platelet count (Table 12).

### 3.10. Studying Multiple Logistic Regression for Serum 25-OHD and Serum Albumin Levels

There was a positive OR for 25-OHD, supporting our theory that the low serum level of 25-OHD is associated with increased severity of the liver disease and HE (Table 13).

## 4. Discussion

Vitamin D deficiency is frequently found in cirrhotic patients. The liver is an essential organ for the biosynthesis of vitamin D, where it is the main organ for the production of 25-OHD [39]. The reduced number of liver cells, decreased exposure to sunlight, greatly reduced adipose tissue mass, impaired vitamin D absorption, and hydroxylation in the liver are all reasons for the low vitamin D levels in cirrhotic patients [49].

We tried in our research to study the relationship between low serum 25-OHD and HE in patients with HCV-related liver cirrhosis and to evaluate whether there is a link between low serum 25OHD and mortality rate.

The study revealed a statistically significant correlation between the severity of cirrhosis (CTP and MELD scores) and low serum levels of 25-OHD. We found that 25OHD levels were negatively correlated with both MELD and CTP scores, suggesting that as the disease advances, 25OHD levels become more deficient (*P* < 0.001). CTP class C contained more 25OHD-deficient cases than class B or class A as the level of vitamin D in the Child A group (53.3 ± 4.4 nmol/L) was higher than in the Child B group (35.9 ± 7.2 nmol/L) while that in the Child B group is higher than in Child C (21.2 ± 9.1 nmol/L) group. Similar results were seen in a study by Fisher et al., in which more vitamin D-deficient cases were found in class C [50].

Moreover, the results of our study were similar to many other studies which have shown that vitamin D level is inversely correlated to the CTP score and MELD [51]. Two studies were conducted by Fernández et al. and Zhao et al. and found that vitamin D decreases further as cirrhosis of the liver becomes more advanced. Thus, patients with higher CTP and MELD scores had notably lower vitamin D levels compared to patients with lower CTP and MELD scores [52, 53].

The study also showed that the serum 25-OHD level was significantly different between the three SBP groups (*P* < 0.001). The serum 25-OHD level was significantly lower in patients with classic SBP (13 ± 3.4 nmol/L) and MNB (13.5 ± 4.9 nmol/L) compared to patients with CNNA (23.5 ± 3 nmol/L, *P* < 0.001).

Vitamin D induces synthesis of LL-37 in human macrophages, which is really the only antibacterial peptide controlled by vitamin D in humans [39]. Notably, insufficient production of LL-37 in the peritoneal cavity induced by vitamin D deficiency in patients with cirrhosis and ascites has been hypothesized to contribute to an increased risk of SBP with increments in the mortality rates [54].

Our study showed that there was a statistically significant difference in vitamin D levels between both groups (*P* < 0.001). As regards the comparison of vitamin D levels in different HE groups, there was a statistically significant difference (*P* < 0.001) as the level of vitamin D in Grade 1 is higher than that in Grade 2 that is higher than that in Grades 3 to 4. This is in agreement with two studies done by Vidot et al. and Iruzubieta who stated that the lower the level of 25-OHD, the higher the HE grade. Deficient anti-inflammatory properties of 25-OHD can explain this association. Systemic inflammation and changes in hepatic metabolism such as elevated levels of ammonia are considered HE precipitants and exacerbate the current HE [55, 56].

In a study done by Stokes et al., it was unclear whether there is a steady decline in brain function as the 25-OHD levels drop or whether there is a threshold at which point there is a significant reduction in brain function in patients with cirrhosis. To show a causative relationship of vitamin D with HE, it was necessary to examine the effects of vitamin D supplementation on encephalopathy symptoms in patients with CLD. The literature suggests that it is an association, not causality. There was a sufficient association between cognitive and behavioral changes associated with 25-OHD deficiency to suggest a role for vitamin D deficiency in the development of HE in patients with liver cirrhosis [41].

In our study, a high mortality rate was associated with serum 25-OHD deficiency. A comparison of vitamin D levels in those who died and those who were improved in the HE group showed that there was a statistically significant difference. Vitamin D level was higher in patients who were improved (35 patients) with a mean 25-OHD level (32.5 nmol/L) in comparison with those who died (15 patients) with a mean 25-OHD level (15.1 nmol/L) and (*P* < 0.001). Because patients with cirrhosis had lower serum 25-OHD levels, we suggested that 25-OHD levels might be of prognostic value in these patients [41, 57, 58]. The ROC analysis showed that the cut-off level of vitamin D > 18.5 nmol/L can predict the survival in HE with a sensitivity of 94% and a specificity of 80% and this is in concordance with a study conducted by Paternostro et al. which reported a high mortality rate in cirrhotic patients with serum 25-OH D levels less than 6 ng/mL (HR 1.723, *P* = 0.013) [59].

Recently, several studies have found the association between serum albumin level and HE and role of human albumin infusion for prevention and treatment of HE [60]. Our study showed that multiple logistic regression for the serum 25-OHD and serum albumin levels in association with HE revealed positive OR, confirming our theory that the low serum level of 25-OHD is associated with increased severity of the liver disease and HE.

Finally, we hope that our study will help physicians understand that maintaining normal vitamin D levels will provide a better prognosis and a better quality of life for CLD patients.

## 5. Limitations

The main limitation of the study is the sample size. A larger study population is essential for the best results.

## 6. Conclusion

Vitamin D deficiency was common in the majority of patients with CLD. Low levels of 25-OHD were associated with HE in patients with HCV-related liver cirrhosis. The worsening vitamin D deficiency was associated with increased severity of liver disease so it may be considered a prognostic parameter for the severity of liver cirrhosis. In addition, it may be linked to a higher mortality rate in the HE group.

## Figures and Tables

**Table 1:** Characteristics and biomarkers in both groups.

	Group 1 (HE)		Group 2 (non-HE)		*P* value
	Mean	SD	Mean	SD	
Age (years)	51.1	6.2	50.6	7.2	0.733
BMI (kg/m^2^)	28.7	3.5	25.7	3.1	<0.001
25-OHD (nmol/L)	27.2	11.9	41.0	15.2	<0.001
Bilirubin (mg/dL)	3.7	1.3	2.2	0.9	<0.001
Creatinine (mg/dL)	1.1	0.5	0.8	0.4	0.001
Albumin (mg/dL)	2.9	0.3	3.5	0.4	<0.001
INR	1.9	0.3	1.2	0.3	<0.001
Sodium (mEq/L)	133.9	3.8	137.4	3.8	<0.001
Potassium (mEq/L)	4.2	0.8	4.1	0.6	0.521
HB (g/dL)	10.3	1.5	11.9	2.0	<0.001
WBC × 10^6^	5.8	2.9	6.5	2.4	0.158
PLT × 10^3^	111.3	30.4	151.3	28.5	<0.001
CTP score	11.8	2.4	7.0	2.1	<0.001
MELD score	21.8	5.6	12.6	5.9	<0.001

Summary of the results of the descriptive study of demographic, laboratory, and clinical variables included in the CTP and MELD scores. BMI: body mass index; 25-OHD: 25-hydroxy vitamin D; INR: international normalized ratio; HB: hemoglobin; WBC: white blood cell count; PLT: platelet count; CTP: Child Turcotte Pugh; MELD: model for end Stage liver disease. The independent *t*-test was used.

**Table 2:** Other characteristics in both groups.

			Groups		*P* value
			1 (HE)	2 (non-HE)	
Sex	Male	*N*	38	33	0.271
		%	76.0%	66.0%	
	Female	*N*	12	17	
		%	24.0%	34.0%	
CTP	A	*N*	0	27	<0.001
		%	0.0%	54.0%	
	B	*N*	13	16	
		%	26.0%	32.0%	
	C	*N*	37	7	
		%	74.0%	14.0%	
Ascites	None	*N*	3	27	<0.001
		%	6.0%	54.0%	
	Medically controlled	*N*	20	13	
		%	40.0%	26.0%	
	Poorly controlled	*N*	27	10	
		%	54.0%	20.0%	
SBP	No	*N*	34	50	<0.001
		%	68.0%	100.0%	
	SBP (classic)	*N*	5	0	
		%	10.0%	0.0%	
	SBP (CNNA)	*N*	8	0	
		%	16.0%	0.0%	
	SBP (MNB)	*N*	3	0	
		%	6.0%	0.0%	
HRS	HRS	*N*	9	0	0.003
		%	18.00%	0.00%	
	No	*N*	41	50	
		%	82.00%	100.00%	
H/M	No	*N*	40	50	0.001
		%	80.0%	100.0%	
	Yes	*N*	10	0	
		%	20.0%	0.0%	
Prognosis	Death	*N*	15	0	<0.001
		%	30.00%	0.00%	
	Improved	*N*	35	0	
		%	70.00%	0.00%	

Summary of the results of the descriptive study of (demographic, laboratory, and clinical variables) included in the CTP and MELD scores. CTP: Child Turcotte Pugh; SBP: spontaneous bacterial peritonitis; HRS: heptorenal syndrome; H/M: hematemesis and/or melena. The chi-square test and Fisher's exact tests were used.

**Table 3:** Comparison of 25-OHD levels between the two groups.

25-OHD level		Groups		*P* value
		1 (HE)	2 (non-HE)	
Insufficient	*N*	0	23	<0.001
	%	0.00%	46.00%	
Mild deficiency	*N*	30	19	
	%	60.00%	38.00%	
Moderate deficiency	*N*	12	5	
	%	24.00%	10.00%	
Severe deficiency	*N*	8	3	
	%	16.00%	6.00%	

The more 25-OHD deficiency was noticed in the HE group. The chi-square test was used.

**Table 4:** Common etiologies of HE in Group 1.

Etiology of HE	*N*	%
SBP	16	32.0%
HRS	9	18.0%
H/M	10	20.0%
Hypokalemia	7	14.0%
Other	8	16.0%
Total	50	100.0

SBP was the most common etiology in the HE group. SBP: spontaneous bacterial peritonitis; HRS: heptorenal syndrome; H/M: hematemesis and/or melena; Other: means other causes of HE-like infections (other than SBP), constipation, diarrhea, or even undetermined causes.

**Table 5:** Comparison of 25-OHD level in different HE etiologies.

	*N*	Mean	SD	*P* value	Comparisons
SBP	16	18.4	6.1	<0.001	SBP & HRS < (H/M) < hypokalemia
HRS	9	16.8	8.2		
H&/M	10	29.3	3.7		
Hypokalemia	7	44.9	2.5		
Other	8	41.6	5.3		

Comparison of 25-OHD levels in patients with HE due to different etiologies. Patients with HE due to SBP and HRS showed the least serum 25-OHD levels followed by 25-OHD levels in HE patients due to H/M and hypokalemia. SBP: spontaneous bacterial peritonitis; HRS: heptorenal syndrome; H&/M: mematemesis and/or melena; Other: means other causes of HE-like infections (other than SBP), constipation, diarrhea, or even undetermined causes. The one-way ANOVA test was used.

**Table 6:** Comparison of 25-OHD level in different SBP categories.

SBP	*N*	Mean	SD	*P* value	Comparisons
SBP(classic)	5	13.0	3.4	<0.001	SBP (classic and MNB) < SBP (CNNA)
SBP(CNNA)	8	23.5	3.0		
SBP(MNB)	2	13.5	4.9		

The levels of 25-OHD in different SBP categories were illustrated. The lowest levels were detected in classic type, followed by MNB and CNNA types. SBP: spontaneous bacterial peritonitis; CNNA; culture-negative neutrocytic ascites; MNB: monobacterial. The Kruskal-Wallis test was used.

**Table 7:** Comparison of 25-OHD levels in different CTP categories.

CTP	N	Mean	SD	P-value	Comparisons
A	27	53.3	4.4	<0.001	A > B > C
B	29	35.9	7.2		
C	44	21.2	9.1		

Comparison of 25-OHD levels in different CTP categories. Levels were decreased from CTP Grade A to Grades B and C. CTP: Child Turcotte Pugh. The one-way ANOVA test was used.

**Table 8:** Correlation between 25-OHD level and MELD score.

		MELD score
25-OHD (nmol/L)	Pearson correlation	-0.909
	*P* value	<0.001
	*N*	100

A highly significant negative correlation between 25-OHD levels and MELD scores was detected. Pearson's correlation test was used.

**Table 9:** Comparison of 25-OHD level in different HE grades.

HE grade	*N*	Mean	SD	*P* value	Comparisons
Grade 1	12	43.8	2.8	<0.001	Grade 1 > Grade 2 > Grades 3 to 4
Grade 2	18	29.6	3.3		
Grade 3 to 4	20	15.3	5.0		

The 25-OHD level is significantly lower in higher grades of HE. The Kruskal-Wallis test was used.

**Table 10:** Comparison of 25-OHD levels in those who died and those improved in the HE group.

Prognosis	*N*	Mean	SD	*P* value
Died	15	15.1	7.1	<0.001
Improved	35	32.5	9.6	

The level of 25-OHD was significantly lower in HE patients who died. The independent *t*-test was used.

**Table 11:** Showing cut-off values of 25-OHD for survival in the HE group.

Coordinates of the curve		
Test result variable(s): 25-OHD (nmol/L)		
Positive if greater than or equal to	Sensitivity	1 − specificity
7.000	1.000	1.000
8.500	1.000	0.933
9.500	1.000	0.867
10.500	1.000	0.733
11.500	1.000	0.600
12.500	1.000	0.467
13.500	1.000	0.400
14.500	1.000	0.333
15.500	1.000	0.267
16.500	0.971	0.267
18.500	0.943	0.200
20.500	0.886	0.200
21.500	0.857	0.200
22.500	0.829	0.200
23.500	0.800	0.200
25.000	0.771	0.200
26.500	0.686	0.200
27.500	0.629	0.133
28.500	0.629	0.067
29.500	0.543	0.000
30.500	0.486	0.000
31.500	0.457	0.000

Cutoff values of 25-OHD for survival in the HE group: cutoff value 18.5 (nmol/L) of 25-OHD level was detected to predict improvement in the HE group with a sensitivity of 94% and a specificity of 80%.

**Table 12:** Correlation between 25-OHD levels and other biochemical parameters in the HE group.

	Bilirubin	Creatinine	Albumin	INR	Sodium	Potassium	HB	WBC	PLT
Pearson correlation	-0.846	-0.55	0.811	-0.653	0.708	-0.39	0.203	-0.166	0.276
P-value	<0.001	<0.001	<0.001	<0.001	<0.001	0.005	0.158	0.249	0.052
N	50	50	50	50	50	50	50	50	50

The table is showing a strong negative correlation in the HE group, between bilirubin level, creatinine, INR, and potassium and serum 25-OHD. A strong positive correlation was detected between albumin level and sodium level and serum 25-OHD level. No correlation was found between 25-OHD level and hemoglobin level, WBCs, or platelet count. Pearson's correlation test was used.

**Table 13:** Multiple logistic regression for the serum 25-OHD and serum Albumin levels in association with HE.

	OR	*P* value	95% CI for OR	
			Lower	Upper
25-OHD (nmol/L)	1.063	<0.002	1.027	1.109
Albumin	12591.328	<0.001	178.757	886908.689

The table is showing multiple logistic regression for the serum 25-OHD levels and serum albumin levels with positive OR, confirming our theory that low serum level of 25-OHD is associated with increased severity of the liver disease and HE.

## Data Availability

The datasets used and analyzed during the current study are available from the corresponding author on reasonable request.
